# Mechanisms of Invasion in Glioblastoma: Extracellular Matrix, Ca^2+^ Signaling, and Glutamate

**DOI:** 10.3389/fncel.2021.663092

**Published:** 2021-06-02

**Authors:** Jae-Seon So, Hyeono Kim, Kyung-Seok Han

**Affiliations:** Department of Medical Biotechnology, Dongguk University-Gyeongju, Gyeongju, South Korea

**Keywords:** glioblastoma, invasion, extracellular matrix, Ca^2+^, glutamate, ion channels

## Abstract

Glioblastoma (GBM) is the most common and malignant form of primary brain tumor with a median survival time of 14–16 months in GBM patients. Surgical treatment with chemotherapy and radiotherapy may help increase survival by removing GBM from the brain. However, complete surgical resection to eliminate GBM is almost impossible due to its high invasiveness. When GBM cells migrate to the brain, they interact with various cells, including astrocytes, neurons, endothelial cells, and the extracellular matrix (ECM). They can also make their cell body shrink to infiltrate into narrow spaces in the brain; thereby, they can invade regions of the brain and escape from surgery. Brain tumor cells create an appropriate microenvironment for migration and invasion by modifying and degrading the ECM. During those processes, the Ca^2+^ signaling pathway and other signaling cascades mediated by various ion channels contribute mainly to gene expression, motility, and invasion of GBM cells. Furthermore, GBM cells release glutamate, affecting migration via activation of ionotropic glutamate receptors in an autocrine manner. This review focuses on the cellular mechanisms of glioblastoma invasion and motility related to ECM, Ca^2+^ signaling, and glutamate. Finally, we discuss possible therapeutic interventions to inhibit invasion by GBM cells.

## Introduction

Gliomas are a common type of primary central nervous system tumors derived from non-neuronal glial cells and include astrocytomas, oligodendrogliomas, and ependymomas. Gliomas are classified into grades I–IV by the World Health Organization (WHO) based on specific pathological features, treatment strategies, and malignancy state (Louis et al., 2007). The higher the WHO grade, the more aggressive the tumor. Glioblastoma (GBM) is a grade IV glioma and the most aggressive and deadliest of primary brain cancers; it arises from astrocytes. The main symptoms of GBM are advancing neurological deficits, persistent headaches, loss of appetite, double or blurred vision, vomiting, and seizures. Of the patients diagnosed with glioma and analyzed in the United States between 2000 and 2014, 61.5% had glioblastomas (Ostrom et al., 2018). The overall survival rate of GBM patients is 39.7% at 1 year and 5.5% at 5 years (Ostrom et al., 2017). Even with medical treatments such as radiation, temozolomide administration, and surgery, the median survival for patients with diagnosed GBM is still only 12–18 months (Stupp et al., 2005; Wen and Kesari, 2008). The highly infiltrative behavior of GBM cells makes it impossible to completely remove the tumor by surgical intervention, causing treatments to be less effective. Furthermore, the invasive nature of GBM results in the destruction of normal brain structures and functions.

Less than 2% of glioblastoma cells migrate beyond the brain (Beauchesne, 2011; Lun et al., 2011; Hamilton et al., 2014), with most GBM cells infiltrating into healthy brain tissue through the perivascular space around blood vessels and the brain parenchyma space that contains neuron and glial cells (Cuddapah et al., 2014). For glioma cells to penetrate, changes in several key factors are needed: energy metabolism (Horing et al., 2012; Kathagen-Buhmann et al., 2016), ion channels (Thompson and Sontheimer, 2016), neurotransmitters, proteases (Demuth and Berens, 2004), cytoskeleton, cell adhesion, and remodeling of the extracellular matrix (ECM) (Cuddapah et al., 2014). The brain ECM is critically involved in various cellular processes, including migration and invasion of glioma cells associated with altering microenvironmental composition (Giese and Westphal, 1996; Charles et al., 2012). Furthermore, intracellular Ca^2+^ signaling through inositol 1,4,5- triphosphate receptors (IP_3_Rs), store-operated channels (SOCs), transient receptor potential (TRP) channels, voltage-gated Ca^2+^ channels (VGCCs), P2 × 7 receptors, and ionotropic glutamate receptors contribute to the motility of glioma cells. This review describes a cellular invasion mechanism of glioblastoma cells that is associated with the ECM and Ca^2+^ signaling.

## The Interaction Between the Extracellular Matrix and Binding Proteins for Migration

The brain ECM forms a physical barrier but actively interacts with the environment through the signaling behavior of a variety of ligands (Miyata and Kitagawa, 2017). Cell migration requires a coordinated process including adhesion of the cell and attachment to and detachment from the ECM (Ridley et al., 2003). The ECM is a highly organized structural network providing structural support and allowing cellular growth, survival, maturation, differentiation, and migration (Theocharis et al., 2016). Glioblastoma cells continuously interact with the ECM, experiencing remodeling for migration and infiltration. Several of the ECM molecules involved in migration are proteoglycans and their binding partners, including hyaluronan, tenascins, glycoproteins, galectins, laminin, and fibrous proteins.

Proteoglycans are heavily glycosylated proteins consisting of core proteins covalently linked to glycosaminoglycan (GAG). Proteoglycans such as heparin sulfate proteoglycan (HSPG) and chondroitin sulfate proteoglycan (CSPG) regulate cellular movement through a variety of signaling pathways. It has been reported that proteoglycan mRNA expression in human GBM is altered compared to that in normal human brain tissue. A subset of GBM highly express CD44, PTPRZ1, and CSPG4/NG2 of the membrane-associated proteoglycans (Wade et al., 2013). CD44 is up-regulated in a GBM dependent on variant form six of the CD44/AKT signaling pathway (Jijiwa et al., 2011). Overexpression of NG2 produces properties similar to those of GBM patients, and knockdown of NG2 using shRNA reduces tumor growth and angiogenesis (Wang et al., 2011). Blocking of PTPRZ1 expression suppresses GBM growth *in vivo* (Ulbricht et al., 2006). One of the most up-regulated ECM constituents is Versican (VCAN), a member of a family of large aggregating CSPGs. The expression level of the VCAN isoform is altered in brain tumor and has been associated with metastasis of gliomas (Paulus et al., 1996). VCAN mediates glioma migration via TGF-β2 signaling, which induces the malignant property of brain tumors (Arslan et al., 2007). Also, VCAN was substantially up-regulated in a cerebral cortex lesion (Asher et al., 2002). Brevican, a major proteoglycan in the adult human brain, is overexpressed in glioma cells and correlates with late-stage tumor metastasis (Dwyer et al., 2014). Brevican promotes glioma cell motility after proteolytic cleavage by ADAMTS4 and the up-regulation of integrin (Held-Feindt et al., 2006; Hu et al., 2008; Lu et al., 2012).

Hyaluronan (HA) is the principal GAG of brain ECM and contains glucuronic acid and *N*-acetyl glucosamine (Park et al., 2008; Amorim et al., 2021). Hyaluronan concentration is high in invasive cancer cells and mediates tumor cell proliferation, migration, and invasion by interacting with CD44 and Receptor for HA-Mediated Motility (RHAMM) receptors (Lokeshwar et al., 1997; Toole, 2004; Lokeshwar et al., 2014). Hyaluronan also facilitates invasion of human glioma cells and the secretion of matrix metalloproteinase (MMP) and plasminogen activator (PA) while not affecting proliferation (Nakagawa et al., 1996). MMP is responsible for invasion and progression through the degradation of ECM components, and MMP-2, MMP-9, MT-MMP levels are significantly increased in human gliomas (Chintala et al., 1999). One of the HA receptors, CD44, binds HA in the extracellular space and cytoskeletal components in the intracellular area, acting as a cell membrane glycoprotein and is involved in numerous cellular processes (Tsukita et al., 1994; Misra et al., 2015; Naor, 2016). CD44 expression is increased in GBM, and it elevates the invasion and proliferation of GBM (Kuppner et al., 1992; Breyer et al., 2000; Anido et al., 2010). CD44-specific antisense oligonucleotide, which prevents CD44 expression, substantially inhibits invasion of glioma cells (Merzak et al., 1994). Inhibition of the interaction between HA and CD44 reduces glioblastoma invasion in hydrogels lacking matrix-bound HA, suggesting of production of HA in GBM cells (Chen et al., 2017). Hyaluronan is synthesized in extracellular region and degraded into different size. Glioblastoma invasiveness is directly affected by HA polymer molecular weight within a methacrylamide-functionalized gelatin (GelMA) hydrogel (Chen et al., 2018). Consistent with these results, the CD44 expression level is higher in severe grade glioma cells, and the anti-CD44 antibody effectively reduces the migration of GBM (Yoshida et al., 2012). Epidermal growth factor stimulation promotes CD44 mRNA expression, which results in glioma cell invasion (Monaghan et al., 2000). The other HA-binding protein is Receptor for HA-Mediated Motility (RHAMM), which has increased expression in higher grade glioma cells, and RHAMM soluble peptide suppresses cell proliferation (Akiyama et al., 2001). Overall, HA interactions with CD44 and RHAMM are critical for tumorigenesis and brain tumor invasion.

Tenascins are large multimeric glycoproteins that are differentially expressed in adults, during embryonic development, and in normal and cancer cells (Latijnhouwers et al., 2000; Orend and Chiquet-Ehrismann, 2006). They are thought to have important roles in the migration and invasion of glioma. Tenascin-C (TN-C) near blood vessels is more highly concentrated in glioblastomas than in low-grade astrocytic tumors (Kim et al., 2000) and is expressed in human glioma *in vivo* (Brosicke et al., 2013). Overexpression of interleukin-33, induced by inflammation, increases invasion of GBM and is associated with elevated TN-C expression via the PI3K and NF-κB signaling pathways (Zhang J. F. et al., 2019). Moreover, TN-C triggers glioma invasiveness through MMP-12 (Sarkar et al., 2006). Additionally, TNIIIA2, a synthetic TN-C peptide, positively regulates the adhesion and migration of cells interacting with integrin (Saito et al., 2007). In the tumor microenvironment, TN-C increases glioblastoma invasion and negatively regulates proliferation (Xia et al., 2016). Tenascin-C also promotes invasion of brain tumor-initiating cells, which is regulated by metalloproteinase ADAM-9 (Sarkar et al., 2015). Consistently, shRNA designed for targeting TN-C impairs glioma cell motility in wound-scratch assay (Angel et al., 2020). It has been suggested that Tenascin-R (TN-R) and Tenascin-W (TN-W), as members of the tenascin family, may be associated with progression and malignancy of glioma cells; however, that hypothesis remains to be elucidated.

**FIGURE 1:**
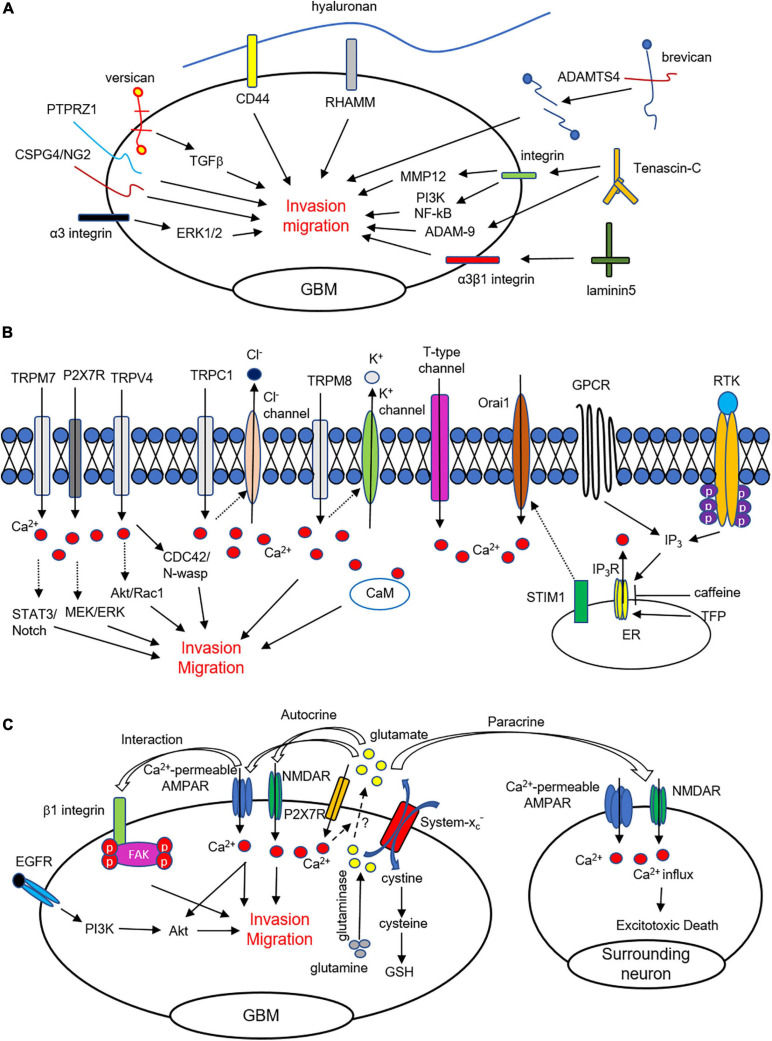
ECM, Ca^2+^ signaling, and glutamate mediates invasion and migration in glioma cells. **(A)** Extracellular matrix and their binding partners regulate invasion and motility in GBM cells. **(B)** Intracellular Ca^2+^ elevation from ER and extracellular region affects glioma cell migration and invasion. Ca^2+^ influx through TRP channels, P2 × 7Rs, and t-type Ca^2+^ channels are critically involved in glioma cell infiltration. **(C)** Glioma cells release glutamate through a cysteine-glutamate exchanger (system x_*C*_^–^). Released glutamate from glioma cells activates Ca^2+^-permeable AMPARs and NMDARs and affects migration and invasion. Sustained Ca^2+^ influx causes excitotoxic death of surrounding cells to make microenvironment for invasion.

Integrins, binding proteins with functions in the infiltration of glioma, stimulate cell adhesion and interact with the ECM as transmembrane heterodimeric receptors composed of α and β chains. In particular, specific antibodies against the αv and β1 integrins (Friedlander et al., 1996) and the treatment of integrin inhibitors (Ishida et al., 2014) suppress glioma cell migration. Inconsistently, it has been suggested that blocking the αv integrin subunit could enhance invasion of glioma in non-migratory glioma (Treasurywala and Berens, 1998). To diagnosis GBM, integrin αvβ3 can be targeted by using hydrocyanines to detect the presence of reactive oxygen species (Zhang L. et al., 2019). Moreover, α3β1 integrins, specific receptors of laminin-5, are highly expressed and have a critical role in the motility and invasion of glioma cells (Fukushima et al., 1998; Zhou et al., 2015). It has also been reported that α3 integrins are critical for the invasiveness of glioma stem-like cells and act through the ERK1/2 pathway (Nakada et al., 2013). Furthermore, signaling mediated by integrins can modulate the activities of MMP and PA to degrade the ECM and allow glioma to invade. Taken together, through their interactions with the ECM, integrins are critically involved in glioma pathogenesis, migration, and infiltration (Figure 1A).

Glioma cells infiltrate the brain area using cell-ECM interactions and the associated dynamics. Proteoglycans, hyaluronan, and tenascins have major roles in the brain ECM during invasion, and the associated binding partners, including CD44, RHAMM, and integrins, also have critical roles. Furthermore, ECM molecules including laminins, reelin, heparin-binding growth-associated molecule, tenascin-R, tenascin-C, and CSPG have been suggested in synaptic plasticity and neuronal activity (Dityatev and Schachner, 2003). Interaction of various ECM components with cell surface recognition molecules, receptors, and ion channels affects synaptic plasticity by regulating Ca2 + influx, signaling in endocytic zones, small GTPases, and ECM remodeling (Dityatev and Schachner, 2003). In C6 glioma cell implanted rats, synaptic plasticity is impaired and neuronal activity is abnormally changed (Wang et al., 2011). Probably, abnormal interaction between ECM and its binding partners could cause long-term memory deficit, one of the symptoms in GBM patients, which could be further tested.

## Calcium Signaling in Glioblastoma Is Related to Migration and Invasion

An accumulation of evidence demonstrates that Ca^2+^ is critical for tumorigenesis in GBM and is related to proliferation, motility, and invasiveness. GBM cells express various receptor tyrosine kinase (RTK) and G protein-coupled receptors (GPCRs) that contribute to Ca^2+^ release from the endoplasmic reticulum (ER). Various agonists for RTK and GPCR increase Ca^2+^ release from the ER via the activation of inositol 1,4,5-trisphosphate receptors (IP_3_Rs) in GBM cells (Kang et al., 2010). Blocking of IP_3_R-mediated Ca^2+^ release by caffeine was shown to inhibit GBM invasion and extend survival in a skin xenograft model injected with GBM cells (Kang et al., 2010). On the other hand, trifluoperazine (TFP) inhibits proliferation, motility, and invasion by largely increasing IP_3_R-mediated Ca^2+^ from the ER in GBM cells (Kang et al., 2017). These results suggest that abnormal responses the either increase or decrease Ca^2+^ release can suppress glioma cell migration and invasion. Overall, appropriate Ca^2+^ dynamics mediated by IP_3_Rs are responsible for the inhibition of metastasis of GBM cells (Figure 1B).

In addition, Ca^2+^ entry from the extracellular region is associated with a cytosolic Ca^2+^ increase, which leads to diverse intracellular signaling and motility in glioma. Store-operated channels (SOCs), Ca^2+^-permeable transient receptor potential (TRP) channels, voltage-gated Ca^2+^ channels (VGCCs), and P2 × 7 receptors mainly contribute to Ca^2+^ influx in glioma cells. SOCs are activated by depleting Ca^2+^ from the ER, sensed by STIM1, and SOC inhibition reduces proliferation and increases apoptosis of GBM cells (Liu et al., 2011). Furthermore, the expression levels of STIM1 and Orai1 are significantly higher in GBM cells than in primary astrocytes, and silencing of STIM1 and Orai1 decreases GBM invasion (Motiani et al., 2013). Besides, the expression of Orai2 is elevated in high-grade glioma, which is an indication of poor survival in GBM patients (Yuan et al., 2019). Consistently, suppression of Ca^2+^ entry by SOCs inhibits glioma cell motility through the actions of proline-rich tyrosine kinase 2 (Pyk2) (Zhu et al., 2014; Zhu et al., 2020), suggesting that Ca^2+^ entry is significantly associated with the metastatic characteristics of GBM cells (Figure 1B).

Previously, it has been reported that TRP channels including TRPC1, TRPC6, TRPM2, TRPM3, TRPM7, TRPM8, TRPV1, and TRPV2 are overexpressed in GBM patients, implying the contribution of TRP channels in the progression of GBM (Alptekin et al., 2015). TRP-canonical1 (TRPC1) is associated with cell proliferation, tumor size, and regulation of Cl^–^ channels during changes to the volume of glioma cells for migration (Bomben and Sontheimer, 2010; Cuddapah et al., 2013). TRPC6, another TRPC family member, regulates hypoxylation and stability of hypoxia-inducible factor-1 (HIF-1) in human glioma cells under hypoxic conditions (Li et al., 2015). In human glioma cells, attenuation of TRPC6 activity inhibits cell growth and arrests the cell cycle (Ding et al., 2010). Additionally, TRPC4 activation results in a large Ca^2+^ influx and enhancement of cell migration in medulloblastoma, a brain cancer arising from cerebellar precursor cells (Wei et al., 2017).

Another family of TRP channels is also directly associated with glioma progression, including migration and invasion of GBM cells. TRP-melastatin7 (TRPM7) promotes migration and invasion of glioma via activation of STAT3 and Notch signaling pathways (Liu et al., 2014), while glioma invasion was reduced in cells transfected with a TRPM7 mutant (Wan et al., 2019). Pharmacological inhibition or siRNA for TRPM7 also reduces migration and invasion in human glioma cells (Leng et al., 2015) and antagonist for TRPM7 reduces various cellular functions such as proliferation, viability, migration, and invasion in both U251/U87 cells (Wong et al., 2020). Furthermore, a TRPM8 agonist increases cytosolic Ca^2+^, subsequently leading to activation of Ca^2+^-activated K^+^ channels that induce glioma cell migration (Wondergem and Bartley, 2009). Consistently, it has been suggested that blocking of TRPM8 can reduce migration and survival in GBM cells (Klumpp et al., 2017). The expression level of TRPM8 is markedly correlated with the invasiveness of glioma cells (Zeng et al., 2019). TRP-vanilloid 4 (TRPV4), another TRP channel subfamily, is abnormally up-regulated in glioma, promoting migration and invasion through AKT/Rac1 signaling (Ou-Yang et al., 2018). Stimulation of TRPV4 increases migration and invasion through the Cdc42/N-wasp axis by regulating cellular protrusions (Yang et al., 2020). Study of various proteins interacting with TRPV2 in GBM may provide possible biomarkers for GBM diagnosis and lead to novel therapeutics (Donate-Macian et al., 2018). Overall, Ca^2+^ entry through TRP channels is associated with cell survival, proliferation, migration, and invasion of brain tumor cells (Figure 1B).

Voltage-gated Ca^2+^ channels (VGCCs) are classified as having high voltage-activated (P/Q-, N-, L-type), intermediate voltage-activated (R-type), or low voltage-activated (T-type) channels. Blocking of T-type VGCCs by endostatin reduces cell proliferation and migration in human GBM cells (Zhang et al., 2012). Another pharmacological blockade using mibefradil and siRNA-mediated knockdown of T-type VGCC reduces cell survival and induces apoptosis through the mTOR/Akt pathway in GBM (Valerie et al., 2013) and GBM stem-like cells (Zhang et al., 2017). In another study, decreased expression of T-type VGCC reduced the proliferation of glioma cells (Panner et al., 2005). More recently, it was shown that mibefradil treatment and silencing of T-type VGCC by shRNA reduces GBM cell survival by apoptosis (Visa et al., 2019). Blocking of P/Q- and N-type VGCC affects glioma progression by inhibiting proliferation and viability in glioma cell lines and increasing astrocytes and microglia in the near tumor region *in vivo* and *in vitro* (Nicoletti et al., 2017) (Figure 1B).

P2 × 7 receptor (P2 × 7R) is associated with ligand-gated cation channels activated by extracellular ATP binding and leading to intracellular Ca^2+^ mobilization. Ca^2+^ signaling mediated by P2 × 7R is related to the malignant characteristics of glioma cells. P2 × 7R is functionally expressed in rat C6 glioma cells, and its activation increases pro-inflammatory factors and cell mobility (Wei et al., 2008). In brains injected with glioma cells, immunostaining of P2 × 7R shows co-localization with tumor cells and microglia, and in a scratch-wound assay, C6 glioma cell migration elevated by P2 × 7R activation is completely blocked by an antagonist for the receptor (Ryu et al., 2011). Consistent with those results, other groups have reported that an agonist for P2 × 7R increases glioma cell migration and proliferation, which is mediated by the MEK/ERK signaling pathway (Ji et al., 2018). Furthermore, activation of P2 × 7R enhances cell death in radiosensitive M059J human glioma cells (Gehring et al., 2012) and GL261 mouse glioma (Tamajusuku et al., 2010). Intriguingly, suppressing P2 × 7R with an antagonist or shRNA promotes cell growth through the up-regulation of EGFR, p-EGFR, HIF-1α, and VEGF in glioma cells (Fang et al., 2013).

Intracellular Ca^2+^ increase via various ion channels regulates motility and invasion through Ca^2+^-activated K^+^ channels upregulated in malignant glioma (Turner et al., 2014). TRAM-34, a specific inhibitor for Ca^2+^-activated K^+^ channels, reduces tumor infiltration of GBM cells implanted in the brain tissue of mice (Ruggieri et al., 2012). Furthermore, calmodulin (CaM) as a regulator of intracellular Ca^2+^ signaling affects GBM invasion by invadopodia formation (Li et al., 2018).

Most of the research in the field of Ca^2+^ signaling of brain tumor cells related to invasion and migration has been conducted only in the past few years, suggesting that there are still many questions to be solved. Ca^2+^ signaling, remodeling of Ca^2+^ signaling, and Ca^2+^-transporting proteins of GBM cells could be all considered in future investigation.

## Glutamate Signaling for Invasion in Glioma Cells

Glutamate, a major excitatory neurotransmitter in the nervous system, has significant roles in the proliferation, growth, and movement of brain tumor cells. Glioma cells have been shown to produce a large amount of glutamate by glutaminase, an enzyme that converts glutamate from glutamine (Yao et al., 2014), and result in a P2 × 7Rs-mediated intracellular Ca^2+^ increase (Strong et al., 2018). Glutamate is released from glioma cells through system x_*C*_^–^, a cysteine-glutamate exchanger, whose expression level is strongly correlated with brain tumor metastasis (de Groot and Sontheimer, 2011; Takeuchi et al., 2013). Rather than glutamate uptake, glioma cells release large amounts of glutamate, subsequently elevating the extracellular glutamate concentration, leading to excitotoxic death in surrounding neurons; thereby generating a space for cell motility (Ye and Sontheimer, 1999; Chung et al., 2005; Sontheimer, 2008; Noch and Khalili, 2009). Furthermore, inhibition of glutamate release using sulfasalazine, a system x_*C*_^–^ blocker, reduces glioma invasion and tumor growth *in vivo* (Lyons et al., 2007).

Glutamate released from glioma cells activates the Ca^2+^-permeable AMPA receptors (AMPARs) expressed in the same cell or neighboring cells, and such releases can induce Ca^2+^ oscillations that are important for cell movement (Lyons et al., 2007). AMPA receptors control glioma cell’s motility through Akt activation and Ca^2+^ signaling, and activation of the Glutamate-AMPAR-Akt pathway contributes to the invasive growth characteristic of glioma (Ishiuchi et al., 2007). Another study has shown that interactions between AMPARs and β1 integrin induce focal adhesion kinase (FAK) autophosphorylation and Rac activation, thereby facilitating glioma migration and invasion (Piao et al., 2009). In addition, blocking of Ca^2+^-permeable AMPARs inhibits migration, while overexpression of Ca^2+^-permeable AMPARs promotes an increase in the number of migratory cells (Ishiuchi et al., 2002). Propofol, a widely used anesthetic, inhibits invasiveness by increasing surface expression of GluA2-containing AMPARs and downregulating system x_*C*_^–^ expression (Wang et al., 2017). Some researchers have suggested that NMDARs are not functionally expressed in glioblastoma cells (Lyons et al., 2007; Stepulak et al., 2009). However, several studies recently demonstrated that stimulation of NMDARs enhances invasion, whereas MK-801 treatment, an activity-dependent antagonist for NMDARs, reduces invasion in human GBM cells (Muller-Langle et al., 2019; Nandakumar et al., 2019). Kynurenic acid, a non-selective antagonist of all ionotropic glutamate receptors, reduces the migration of human GBM cells (Walczak et al., 2014). Even though metabotropic glutamate receptors 3 and 5 (mGluR3 and mGluR5) are expressed in glioma cells (Condorelli et al., 1997; Stepulak et al., 2009), their roles in cell mobility have not been elucidated. In addition to invasion and migration, glutamate stimulates tumor growth, proliferation, and survival of glioma cells through the EGFR-phospho-Akt and PI3K/AKT pathways (Schunemann et al., 2010; Prickett and Samuels, 2012). Overall, glioma cells release glutamate, which directly affects migration and invasion through the functional expressions of AMPARs and NMDARs in the cell (Figure 1C).

Glutamate and its receptors might be a therapeutic target for the inhibition of GBM invasion through the interaction between tumor cells and nearby cells. GBM cells release not only glutamate but also various molecules including cytokines, metabolites, and nucleic acids contributing to tumor cell progression (Almiron Bonnin et al., 2018). Therefore, targeting the secretory mechanisms could potentially develop therapeutics to reduce GBM invasion.

## Conclusion

**FIGURE 2:**
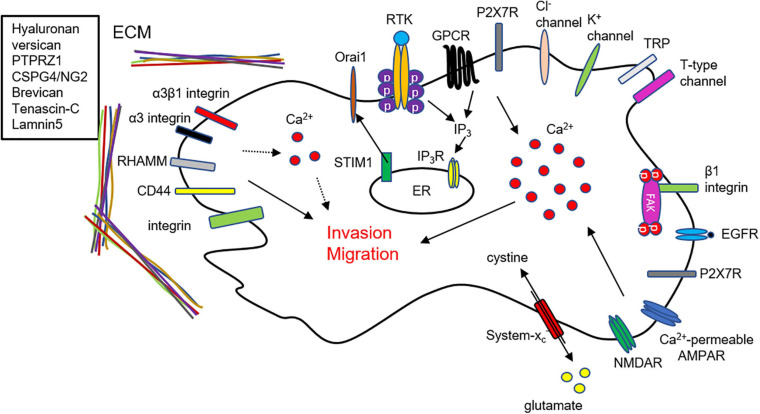
Schematic linking mechanisms of ECM, Ca^2+^ signaling, and glutamate in GBM invasion. Summary of mechanisms of glioma invasion related to ECM, Ca^2+^ signaling, and glutamate. Glutamate release from GBM cells activates Ca^2+^ permeable AMPARs and NMDARs in an autocrine manner, thereby cause an intracellular Ca^2+^ increase in GBM cells. ECM and their binding partners affect GBM invasion by regulating Ca^2+^ signaling.

Invasion is the main characteristic of malignant glioma and one of the obstacles to radiotherapeutic, chemotherapeutic, and surgical treatments. This review provides an overview of ECM, Ca^2+^ signaling, and glutamate release and their associations with glioma cell invasion and migration. These components are linked with each other for invasion and migration in GBM cells (Figure 2). Glutamate released from GBM cells activates Ca^2+^-permeable AMPAR in an autocrine manner contributing to intracellular Ca^2+^ increase for invasion and migration (Figure 2).

To date, current treatment for GBM patients is the surgery to remove the brain tumor, followed by a combination of radiotherapy and temozolomide treatment (Stupp et al., 2005). Other agent bevacizumab that received approval for GBM treatment has shown only moderate effect (Kreisl et al., 2009). In addition, therapeutic interventions targeting ECM, Ca^2+^ signaling, and glutamate and aimed at blocking invasion have not been very successful in GBM patients. Mibefradil, a selective T-type Ca^2+^ channel blocker, followed by temozolomide was given to high-grade glioma patients (Holdhoff et al., 2017). A combination of radiation therapy with cilengitide, an inhibitor for α V integrins, was tested in GBM patients (Eisele et al., 2014). In preclinical studies, matrix metalloproteinase (MMP) inhibitors were effective in the reduction of glioma invasion, but not in clinical trials (Tonn et al., 1999; Koutroulis et al., 2008). Several drugs targeting the PI3K/Akt pathway were partially effective in preclinical studies (Drappatz et al., 2009). Talampanel, the allosteric inhibitor of AMPARs, is used in phase II trial with chemotherapy or radiotherapy (Grossman et al., 2009; Iwamoto et al., 2010).

However, these kinds of therapeutic approaches moderately increase survival and tumor still recur in all cases. A combination of those pharmacological approaches could be worthwhile to attempt, but it remains a great challenge. Glioblastoma can move into normal brain tissue escaping from surgery and radiotherapy due to its high invasiveness. Therefore, better understanding the cellular mechanism of GBM invasion could help to develop a new treatment that suppresses invasion and migration. Furthermore, the therapeutics based on mechanisms that only focus on glioma cells still have limitations. Invasion and migration can be achieved by the complex interplay between glioblastoma and surrounding cells. Therefore, future studies should focus not only on glioma cells but also on the interaction between brain tumor cells and other surrounding cells, including neurons and glial cells.

## Author Contributions

J-SS and K-SH wrote the manuscript and contributed to editing and proofreading the manuscript. HK wrote the glutamate part of the manuscript and prepared the figures. All authors contributed to the article and approved the submitted version.

## Conflict of Interest

The authors declare that the research was conducted in the absence of any commercial or financial relationships that could be construed as a potential conflict of interest.
